# ZKSCAN5 Activates *VEGFC* Expression by Recruiting SETD7 to Promote the Lymphangiogenesis, Tumour Growth, and Metastasis of Breast Cancer

**DOI:** 10.3389/fonc.2022.875033

**Published:** 2022-05-05

**Authors:** Jingtong Li, Zhifeng Yan, Jianli Ma, Zhong Chu, Huizi Li, Jingjing Guo, Qingyuan Zhang, Hui Zhao, Ying Li, Tao Wang

**Affiliations:** ^1^ Department of Medical Oncology, Harbin Medical University Cancer Hospital, Harbin, China; ^2^ Department of Obstetrics and Gynecology, Seventh Medical Center of Chinese People’s Liberation Army (PLA) General Hospital, Beijing, China; ^3^ Department of Radiation Oncology, Harbin Medical University Cancer Hospital, Harbin, China; ^4^ Department of Nutrition, People’s Liberation Army (PLA) Rocket Force Characteristic Medical Center, Beijing, China; ^5^ Department of Oncology, Fourth Medical Center of Chinese People’s Liberation Army (PLA) General Hospital, Beijing, China; ^6^ Department of Oncology, Fifth Medical Center of Chinese People’s Liberation Army (PLA) General Hospital, Beijing, China

**Keywords:** VEGFC, lymphangiogenesis, breast cancer, proliferation and metastasis, SETD7, ZKSCAN5

## Abstract

The growth of lymphatic vessels (lymphangiogenesis) plays a pivotal role in breast cancer progression and metastasis and the immune response. Vascular endothelial growth factor C (VEGFC) has been demonstrated to accelerate cancer metastasis and modulate the immune system by enhancing lymphangiogenesis. However, it remains largely unclear how transcription factors physically regulate VEGFC expression by interacting with histone-modifying enzymes. Like many histone-modifying enzymes, SETD7 plays a key role in cell proliferation and inhibits tumour cell differentiation. In this study, we identified the role of the transcription factor zinc finger with KRAB and SCAN domains 5 (ZKSCAN5) in interacting with histone methyltransferase SETD7 and mediating VEGFC transcription and tumour lymphangiogenesis. ZKSCAN5 interacts with and recruits SETD7 to the VEGFC promoter. By regulating breast cancer-secreted VEGFC, ZKSCAN5 could induce the tube formation of lymph endothelial cells, which promotes tumour proliferation, migration, and metastasis. Clinically, the expression of ZKSCAN5 was frequently upregulated in patients with breast cancer and positively correlated with the expression of VEGFC and the number of lymphatic microvessels. ZKSCAN5 is a poor prognostic factor for patients with breast cancer. Our results characterise the role of ZKSCAN5 in regulating VEGFC transcription and predict ZKSCAN5 as a breast cancer therapeutic target.

## Introduction

Breast cancer is the leading cause of death among women worldwide (1, 2). Lymphangiogenesis, a pivotal component for tumour metastasis, immune escape, and growth, has frequently been shown to occur in human breast cancer (3). Vascular endothelial growth factor C (VEGFC), a member of the VEGF family, is an important regulator of lymphangiogenesis (4–6). VEGFC stimulates the formation of new lymph vessels and provides a route for detached cancer cells to metastasise to distant sites (7). Numerous experiments have demonstrated that tumour-secreted VEGFC is a key cytokine involved in tumour development and the immune response (8, 9). A high VEGFC level is associated with significantly decreased overall and disease-free survival in many solid tumours (10, 11). Thus, VEGFC appears to be an attractive therapeutic target for cancers. Thus, discovering novel factors that regulate VEGFC expression is of great significance.

The transcriptional regulation of VEGFC is one of the most significant ways to control VEGFC expression (12). A small number of transcription factors have been reported to regulate VEGFC expression at the mRNA level, including Six1 (13) and forkhead box k1 (FOXK1) (14). However, novel VEGFC transcriptional factors controlling the VEGFC mRNA level remain largely unknown.

Zinc finger with KRAB and SCAN domains 5 (ZKSCAN5) is a transcription factor that belongs to one of the Krűppel-like zinc finger family members. ZKSCAN5 is pivotal in the process of spermatogenesis (15). It has been validated that ZKSCAN5 is closely linked with oesophageal squamous cell carcinoma tumorigenesis (16). However, the biological functions of ZKSCAN5 remain largely unknown. SETD7 interacts with and methylates a large number of transcription factors, such as BRG1 (17), E2F1 (18), and SMAD3 (19). SETD7-mediated methylation could facilitate the recruitment of transcription factors to chromatin (20, 21).

In this study, we found that ZKSCAN5 interacts with SETD7 and increases VEGFC transcription by facilitating the recruitment of the ZKSCAN5/SETD7 complex to the VEGFC promoter. In addition, ZKSCAN5 has been recognised as a novel critical regulator for the expression of VEGFC and contributes to tumour lymphangiogenesis. ZKSCAN5 promotes the proliferation, migration, and tube formation of human lymphocyte endothelial cells (HLECs). Furthermore, ZKSCAN5 is positively correlated with the expression of VEGFC and could be a valuable prognostic marker for poor survival of breast cancer.

## Materials and Methods

### Plasmids, Antibodies, siRNAs, and Reagents

PCR-amplified fragments were inserted into pGEX-KG (Amersham Pharmacia Biotech, Amersham, UK) or pET-28a (Novagen) to produce plasmids expressing fusion proteins of GST or His. The FLAG-tagged ZKSCAN5 and SETD7 as well as the MYC-tagged ZKSCAN5 and SETD7 eukaryotic expression vectors were constructed by cloning PCR-amplified sequences into pcDNA3 (Invitrogen, Carlsbad, CA, USA). The luciferase reporters of the VEGFC promoter were constructed by cloning promoter DNA fragments obtained from genomic DNA into the pGL4-Basic vector (Promega, Madison, WI, USA).

Anti-Flag (A8592), anti-GAPDH (G9295), anti-Flag M2 agarose (A2220), anti-ZKSCAN5 (SAB4501021), and anti-SETD7 (SAB1306218) antibodies were obtained from Sigma-Aldrich (St. Louis, MO, USA); anti-Myc (sc-40HRP) antibody was obtained from Santa Cruz Biotechnology (Dallas, TX, USA); anti-H3K4me2 (17–677) and anti-H3K4me3 (17–678) antibodies were obtained from Millipore (Burlington, MA, USA); anti-H3K4me (ab8895), anti-SETD7 (ab14820), anti-VEGFC (ab83905), and anti-LYVE1 (ab10278) antibodies were obtained from Abcam (Cambridge, MA, USA); anti-SET1 (A300-289A) and anti-mixed lineage leukaemia protein 1 (MLL1; A300-374A) antibodies were obtained from Bethyl (Montgomery, TX, USA); and anti-His (27471001) and anti-GST (RPN1236) antibodies were obtained from GE Healthcare Life Sciences (Chicago, IL, USA).

The sequences of ZKSCAN5 and SETD7, both short hairpin RNAs (shRNAs) and siRNAs, are provided in **Supplementary Table S1**. A lentiviral pSIH-H1-Puro vector was used to express shRNAs, and stable cell lines were generated using lentiviral transduction (System Biosciences, Palo Alto, CA, USA). siRNAs were chemically synthesised (GenePharma, Shanghai). (R)-PFI-2 (HY-18627A) was obtained from MedChemExpress (Princeton, NJ, USA). GSK-LSD1 2HCL (S7574) and CPI-455 HCL (S8287) were obtained from Selleck (Houston, TX, USA).

### Cell Culture, Transfection, and Luciferase Reporter Assay

Human embryonic kidney 293T cells, breast cancer cells ZR75-1 (ER+), and MDA-MB-231 (ER-) were purchased from ATCC and cultured in DMEM (Invitrogen) with 10% FBS (HyClone, Logan, UT, USA). Lipofectamine 2000 Reagent (Invitrogen) was used for transfection. Integration of lentiviruses was achieved by co-transfecting recombinant lentivirus vectors and pPACK Packaging Plasmid Mix (System Biosciences) into 293T cells using the MegaTran Reagent (OriGene, Rockville, MD, USA). Stable cell lines were kept for approximately 2 months in 1 μg/ml puromycin. The Dual Luciferase Reporter Assay System from Promega was used to perform luciferase reporter assays.

### Screening for Transcription Factors Regulating the VEGFC Promoter

High-throughput screening assays were performed according to the manufacturer’s instructions (OriGene). In brief, screening assay reagents were added to each 384-well plate containing VEGFC-Luc reporter vector (100 ng), galactosidase reporter (100 ng), and distinct cDNA plasmids (60 ng). The mixture was kept at room temperature for 20 min until complex formation, and ZR75-1 cells were added at a density of 7,500 cells/well. After 48 h of incubation, the cells were collected, and subsequently, luciferase activities were analysed.

### Real-Time Reverse Transcription-PCR

Cellular RNA was isolated by using the TRIzol reagent (Invitrogen). Using the Quantscript RT Kit (Promega), reverse transcription of the extracted RNA into cDNA was performed. The relative expression of VEGFC was normalised to β-actin expression. The primers used for quantitative real-time reverse transcription (qRT-PCR) were as follows: VEGFC-forward: 5′-CTCGGATGCTGGAGATGAC-3′, VEGFC-reverse: 5′-GGCTGGGGAAGAGTTTGTT-3′.

### Wound Healing Assays

A micropipette tip was used to scrape the cells in a six-well plate. The cells were cultured in ZKSCAN5-related conditioned medium. Cell migration was monitored and imaged with a microscope at the indicated times. The cell migratory abilities were recorded and analysed with ImageJ software.

### Tube Formation Assay

We placed the thawed extracellular matrix (ECM) gel solution into 96 prechilled sterile well plates, and then they were incubated for 1 h at 37°C to allow the matrix solution to solidify. Cell suspensions of 1.5–3 × 10^4^ cells/well were added to the cured ECM gel. The cells were incubated at 37°C for 6–18 h. An inverted microscope was then used to observe and photograph the tube formation.

### GST Pull-Down and Coimmunoprecipitation Assays

Purified His or GST fusion proteins bound to GST beads supplemented with protease inhibitors were co-incubated at 4°C for 4 h. After washing, the precipitated components were subjected to Western blot analysis. Cells were harvested and lysed using sonication to perform a coimmunoprecipitation assay. The supernatant of the cell lysates was incubated with antibodies at 4°C overnight, followed by incubation with Protein A Agarose (Santa Cruz) at 4°C overnight. The beads were dissolved in 2× SDS loading buffer after washing thrice with lysis buffer washing. Western blot was performed using specific antibodies as indicated.

### Chromatin Immunoprecipitation and Re-ChIP

A Magna ChIP Test Kit (Millipore, Burlington, MA, USA) was used for chromatin immunoprecipitation (ChIP) determination according to the manufacturer’s instructions. Briefly, 1 × 10^7^ ZR75-1 cells were cross-linked with 1% formaldehyde (Sigma) at room temperature, and then 0.25 M glycine was added after 10 min. Chromatin was sonicated to a size range of 200–1,000-bp fragments for ChIP analysis. The primary immunoprecipitation complexes were washed, eluted with 10 mM DTT at 37°C for 30 min, and diluted to 1:50 in re-ChIP buffer followed by re-ChIP with the secondary antibodies. Real-time PCR was conducted to detect the relative mRNA expression. **Supplementary Table S2** summarises the primers used for quantitative real-time PCR analysis.

### *In Vivo* Tumour Growth and Metastasis Analysis

The animal study was approved and monitored by the Ethics Committee of Harbin Medical University Cancer Hospital (the ID of animal experiment ethical approval: SYDW2021-056). For *in vivo* tumour estimation, nude mice were inoculated subcutaneously with 1 × 10^7^ ZR75-1 cells with different constructs on the right side. The tumour size was calculated, and the mice were euthanised at the indicated time. The resected tumour was preserved in liquid nitrogen.

BALB/c mice were injected with 1 × 10^6^ MDA-MB-231 cells labelled with luciferase carrying the indicated constructs into the lateral tail vein. All mice were euthanised after 50 days. All lungs were excised for metastatic foci analysis.

### Immunohistochemistry

Primary breast cancer tissues and adjacent normal tissues were obtained from 116 patients at the Harbin Medical University Cancer Hospital (the ID of clinical experiment ethical approval: SYLC2021-063). Informed consent was obtained from the patients, and all study protocols were approved by the Institutional Review or Committees of Harbin Medical University Cancer Hospital. Anti-ZKSCAN5 (SAB4501021), anti-VEGFC (ab83905), and anti-LYVE1 (ab10278) primary antibodies were used at 1:100, 1:100, and 1:50 dilutions, respectively. The H-score of ZKSCAN5 or VEGFC was calculated by multiplying the percentage of positive cells and staining intensity.

### Statistical Analyses

Statistical significance was assessed by using the two-tailed Student’s *t*-tests. The correlation expression and clinicopathologic characteristics were determined using the Pearson’s χ^2^ tests. The Kaplan–Meier method was used to estimate the overall and disease-free survival. All calculations were conducted with the SPSS 20.0 software. p < 0.05 was considered to indicate statistical significance.

## Results

### ZKSCAN5 Mediates the Transcription of VEGFC in Breast Cancer Cells

**Figure 1:**
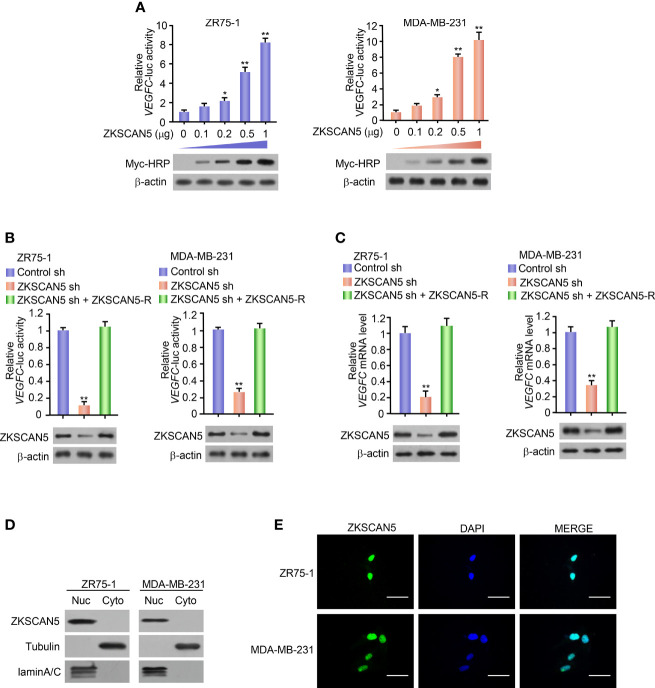
ZKSCAN5 regulates the expression of *VEGFC* in breast cancer cells. **(A)** Luciferase reporter genes were determined in ZR75-1 and MDA-MB-231 breast cancer cells co-transfected with different concentrations of *VEGFC* reporter and myc-ZKSCAN5. A representative immunoblot showed the expression of myc-HRP. β-Actin was used as a control for loading. All values shown are expressed as the average value ± SD obtained from three independent experiments. *p < 0.05, **p < 0.01, and empty vector. **(B)** Luciferase reporter gene detection in ZR75-1 and MDA-MB-231 breast cancer cells co-transfected with *VEGFC*-Luc and ZKSCAN5 shRNA, *VEGFC*-Luc and control shRNA, or *VEGFC*-Luc and ZKSCAN5 shRNA plus shRNA-resistant ZKSCAN5 (ZKSCAN5-R). The representative Western blot shows ZKSCAN5 expression. Among them, β-actin was used as the loading control. **(C)** Real-time RT-PCR was used to analyse the *VEGFC* expression in ZR75-1 and MDA-MB-231 cells, which were transfected with ZKSCAN5 shRNA, control shRNA, or ZKSCAN5 shRNA plus shRNA-resistant ZKSCAN5 (ZKSCAN5-R). The representative Western blot further showed the expression of ZKSCAN5. Data shown are the mean ± SD of triplicate measurements from experiments that have been repeated three times with similar results **(B, C)**. **p < 0.01 versus control shRNA. **(D)** Cytoplasmic and nuclear ZKSCAN5 protein levels in two types of breast cancer cell lines, ZR75-1 and MDA-MB-231. Tubulin was used as the cytoplasmic control, and lamin A/C was used as the nuclear protein-loading control. **(E)** Immunofluorescence images of ZKSCAN5 cellular localisation in green, and nuclei stained in blue (DAPI).

To determine the possible transcription factors regulating VEGFC transcription, we selected a transcription factor from the full-length cDNA transfection array of zr75-1 breast cancer cells from −1,058 to +1 bp by using the VEGFC promoter-luciferase (VEGFC-Luc) reporter. Besides the previously reported transcription factor Six1, we identified a novel transcriptional factor, i.e., ZKSCAN5. With an increase in ZKSCAN5 expression vector transfection doses, the VEGFC-Luc reporter activity gradually increased in both ZR75-1 and MDA-MB-231 cells (**Figure 1A**). By contrast, the knockdown of ZKSCAN5 decreased VEGFC-Luc reporter activity (**Figure 1B**). In accordance with the results of luciferase reporter analysis, knockdown of ZKSCAN5 decreased the VEGFC mRNA level (**Figure 1C**). Since the subcellular localisation of ZKSCAN5 has not been reported, we investigated the subcellular localisation of ZKSCAN5 by performing cytosolic–nuclear separation and immunofluorescence assay. The results showed that ZKSCAN5 was mainly located in the nucleus, which provided the cellular basis of ZKSCAN5 to transcriptionally regulate VEGFC expression (**Figures 1D, E**).

### ZKSCAN5-Regulated VEGFC Promotes the Proliferation, Migration, and Tube Formation of HLECs

**Figure 2:**
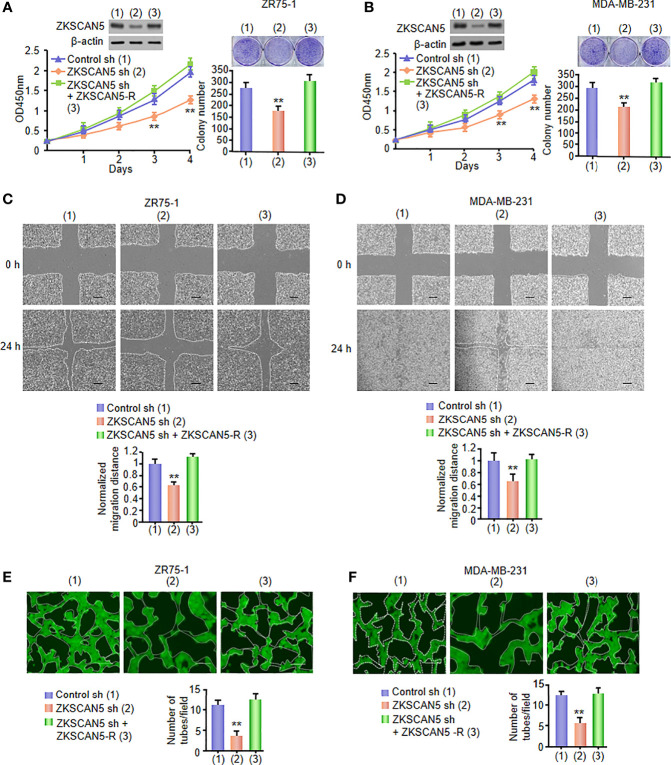
VEGFC secreted by cancer cells, under the influence of ZKSCAN5, regulates HLEC proliferation, migration, and tube formation. **(A, B)** Cell proliferation and colony formation assays in HLECs cultured in conditioned medium come from ZR75-1 or MDA-MB-231 cells stably infected with lentivirus carrying ZKSCAN5 shRNA or ZKSCAN5 shRNA plus ZKSCAN5-R. The representative Western blot displays the expression of ZKSCAN5. **p < 0.01 versus the control shRNA group **(A, B)**. **(C, D)** Wound healing assays for HLECs cultured in conditioned medium from ZR75-1 or MDA-MB-231 cells, which were stably infected as in (A). The image shown is one of the representative results **(C, D)**. Scale bar: 100 μm. **(E, F)** Tube formation assays for HLECs cultured in the conditioned medium from ZR75-1 or MDA-MB-231 cells, which were stably infected as in **(A)**. All values shown are the mean ± SD of triplicate measurements and were repeated three times with analogous results **(C, D)**. *p < 0.05 versus control shRNA. **p < 0.01 versus control shRNA.

Cancer cell-secreted VEGFC markedly enhanced the proliferation and migration of lymphocyte endothelial cells. Because ZKSCAN5 improved the secretion of VEGFC by breast cancer cells, the effects of the conditioned medium on HLEC proliferation and migration were investigated in ZKSCAN5 knockdown stable cell lines. The ZKSCAN5 knockdown ZR75-1 or MDA-MB-231 cell-conditioned medium decreased HLEC proliferation. The conditioned medium from these cells re-expressing ZKSCAN5 could rescue these effects (**Figures 2A, B** **)**. A similar tendency was also detected in HLEC migration analysis (**Figures 2C, D** **)**.

The evolution of capillary lymph ducts by lymphatic endothelial cells is the key aspect of lymphangiogenesis. Therefore, we examined whether the expression of ZKSCAN5-mediated VEGFC could affect HLEC tube formation *in vitro*. The conditioned medium of ZKSCAN5 knockdown breast cancer cells constrained tube formation, which could be rescued by ZKSCAN5 re-expression in the ZKSCAN5 knockdown cells (**Figures 2E, F** **)**. Collectively, these results illustrate that ZKSCAN5 enhances the expression of VEGFC and promotes HLEC tube formation and lymphangiogenesis.

### ZKSCAN5 Regulates Breast Cancer Tumour Growth and Lung Metastasis *In Vivo*

**Figure 3:**
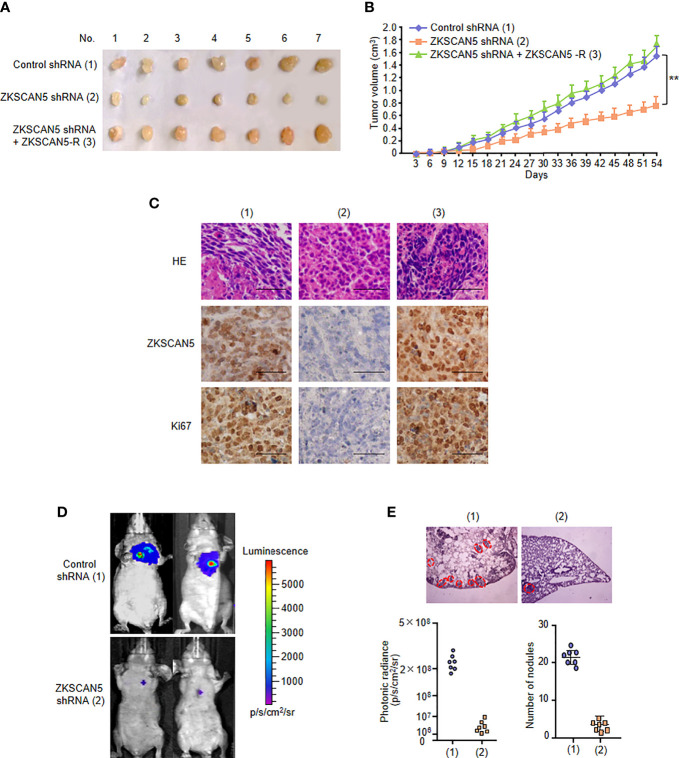
ZKSCAN5 regulates the growth of breast cancer tumours and lung metastasis *in vivo*. **(A, B)** ZR75-1 cells stably infected with the lentivirus carrying the indicated constructs were injected subcutaneously into the nude mice (n = 7 per group). The tumour volume was measured every 3 days, and the growth curve was plotted **(B)**. **(C)** Representative IHC staining of ZKSCAN5 and Ki67 and H&E staining images of tumours resected from nude mice. Scale bar, 50 µm. **(D)** MDA-MB-231 cells stably expressing the constructs were injected through the tail vein to construct a breast cancer cell metastasis model in nude mice (n = 7 per group). **(E)** Anatomical and histological analyses of representative lung metastases were carried out. The number of tumour tubercles was determined under an anatomical microscope. Symbols represent individual mice. **p < 0.01 versus the corresponding control.

To determine the phenotype of ZKSCAN5 *in vivo*, we examined the effect of ZKSCAN5 on breast cancer growth by injecting breast cancer cells containing this structure into the back of BALB/C nude mice. As expected, ZKSCAN5 knockdown significantly inhibited the growth of breast cancer tumours. This could be rescued by ZKSCAN5 re-expression in the ZKSCAN5 knockdown cells (**Figures 3A–C**).

Since metastases occur in about 10% of patients with breast cancer, and nearly half of distant metastases occur in the lungs, we investigated the effect of this pathway on breast cancer tumour metastasis. Compared with that in the control group, diffuse pulmonary nodules were significantly reduced in the ZKSCAN5 knockout group. Importantly, ZKSCAN5 re-expression in the ZKSCAN5 knockdown cells dramatically rescued lung metastasis (**Figures 3D, E** **)**. A histological examination of the lungs confirmed the presence of metastases. In conclusion, ZKSCAN5 regulates breast cancer tumour growth and lung metastasis *in vivo*.

### ZKSCAN5 Recruits the Histone Methyltransferase SETD7 to the VEGFC Promoter

**Figure 4:**
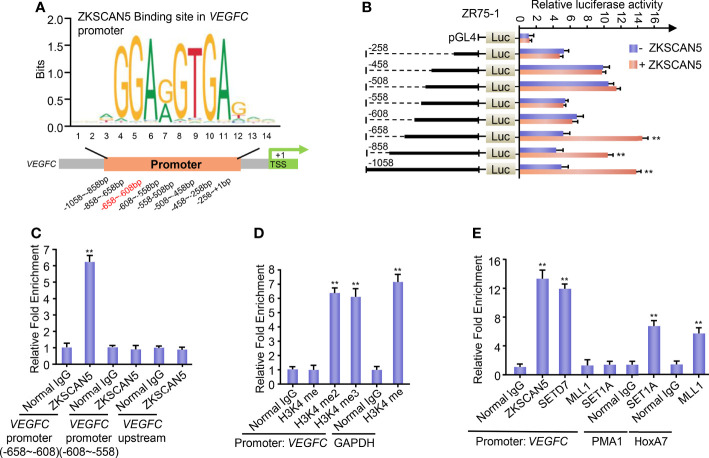
**(A)** Conserved binding sequences of the transcription factor ZKSCAN5 (JASPAR: http://jaspar.genereg.net/) and its binding sites to the VEGFC promoter. **(B)** Luciferase activity of various VEGFC promoter constructs in ZR75-1 cells transfected with ZKSCAN5 or empty vector. Data shown are the mean ± SD of triplicate measurements and were repeated three times with similar results. **p < 0.01 versus empty vector with corresponding promoter reporter. **(C)** ChIP analysis of the occupancy of ZKSCAN5 on the putative ZKSCAN5-binding sites of the *VEGFC* promoter in ZR75-1 cells. **(D)** ChIP analysis of the occupancy of H3K4me, H3K4me2, and H3K4me3 on the *VEGFC* promoter in ZR75-1 cells. The GAPDH promoter has the function of H3K4me2- and H3K4me3-positive control. **(E)** ChIP analysis of the occupancy of ZKSCAN5 and different histone methyltransferases on the *VEGFC* promoter in ZR75-1 cells. Positive controls of SET1 and MLL1 were promoters of PMA1 and HoxA7, respectively.

To further investigate the transcription mechanisms of ZKSCAN5 on regulating VEGFC expression in breast cancer cells, we confirmed the binding site of ZKSCAN5 on the VEGFC promoter. We used JASPAR to predict conserved binding sequences of ZKSCAN5 and its binding sites to the VEGFC promoter (**Figure 4A**). A luciferase assay demonstrated that nucleotides from −658 to −608 bp on the VEGFC promoter contained a possible ZKSCAN5-binding site (**Figure 4B**). ChIP assay revealed that ZKSCAN5 was specifically recruited into the −658- to −608-bp region of the VEGFC promoter, and not the −608- to −558-bp region or 2 kb upstream of the VEGFC promoter (**Figure 4C**).

Transcriptional activation or repression can be led by histone methylation. Methylation of histone H3 at lysine 4 (H3K4) is supposed to be a transcriptional activating mark. Given that ZKSCAN5 benefits VEGFC transcription, we then investigated whether H3K4 methylation enriched the ZKSCAN5-binding region. The specificity of the H3K4 methyl antibodies was validated before the ChIP assay. As expected, GSK-LSD1 (200 μM, 12 h), an LSD1 inhibitor, specifically increases the levels of H3K4me2 and H3K4me3 but does not affect H3K4me1. CPI-455 (10 μM, 5 days), the inhibitor of KDM5 demethylases, only increases the level of H3K4me3 but does not affect H3K4me2. These findings demonstrate that the H3K4methyl antibodies used in our experiments are specific without cross-reactivity (**Supplementary Figure S1**). H3K4 dimethylation (H3K4me2) and trimethylation (H3K4me3), but not H3K4 monomethylation (H3K4me), were enriched at the −658- to −608-bp region, despite the positive control H3K4me being enriched at the GAPDH promoters (**Figure 4D**).

**Figure 5:**
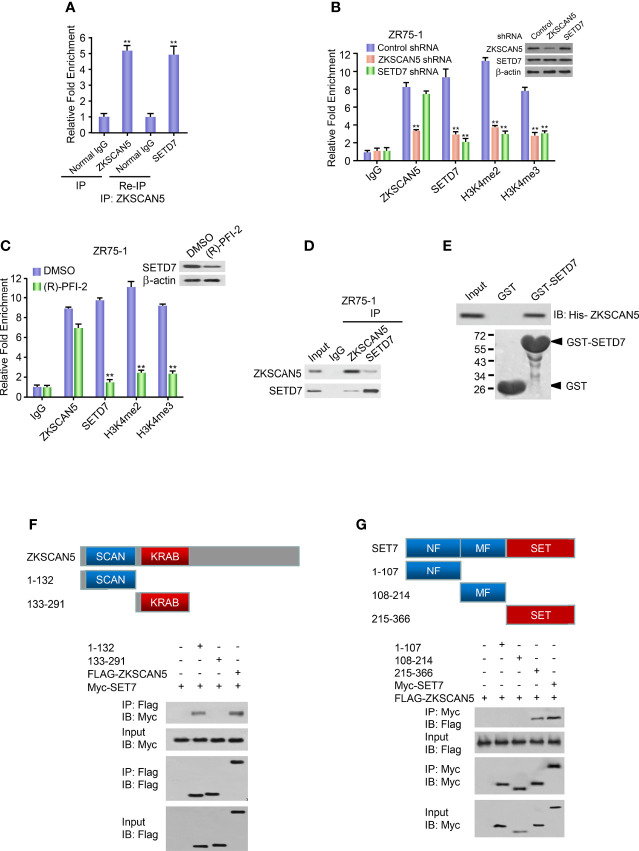
ZKSCAN5 and SETD7 constructed a complex on the −658- to −608-bp region of the VEGFC promoter. **(A)** Re-ChIP analysis of the occupancy of ZKSCAN5 and SETD7 on the VEGFC promoter (−658 to −608 bp) in ZR75-1 cells. **(B)** ChIP analysis of ZR75-1 cells stably infected with lentivirus carrying ZKSCAN5 shRNA or SETD7 shRNA on VEGFC promoter (−658 to −608 bp) with the indicated antibodies. Western blot revealed the knockdown effects of ZKSCAN5 and SETD7. **p < 0.01 versus corresponding control shRNA. **(C)** ChIP analysis using the SETD7 antibody in ZR75-1 cells treated with DMSO or (R)-PFI-2 on the VEGFC promoter (−658 to −608 bp). **(D)** Reciprocal coimmunoprecipitation analysis of endogenous interactions among ZKSCAN5 and SETD7. **(E)** GST pull-down analysis of direct interactions between ZKSCAN5 and SETD7. Purified His-tagged ZKSCAN5 and GST-SETD7 or GST was used. **(F)** Mapping of the interaction region of SET7 in ZKSCAN5. HEK293T cells were co-transfected with MYC-tagged SET7 and FLAG-tagged ZKSCAN5 or its deletion mutants. Anti-FLAG immunoprecipitation was used to precipitate cell lysates, followed by immunoblotting with the specified antibody. The schematic diagram shows ZKSCAN5 and its deletion mutants. **(G)** The mapping highlights the interaction region of ZKSCAN5 in SET7. HEK293T cells were co-transfected with MYC-tagged ZKSCAN5 and FLAG-tagged SET7 or its deletion mutants. Immunoprecipitation of the cell lysate was analysed in **(A)**. The schematic diagram shows SET7 and its deletion mutants; MF, middle region fragment; SET, SET domain-containing fragment. All values shown are the mean ± SD of triplicate measurements from experiments that have been repeated three times with similar results.

Next, we investigated which histone methyltransferase precisely regulates the dimethylation or trimethylation of H3K4 on the ZKSCAN5-binding region (−658 to −608 bp). Like ZKSCAN5, SETD7 was also recruited to the ZKSCAN5-binding site on the VEGFC promoter (**Figure 4E**). As previously reported (22, 23), although MLL1 and SET1A were recruited to the promoters of homeobox-containing 7 and plasma membrane ATPase 1 separately, they were not recruited to the binding site of the VEGFC promoter (**Figure 4E**). Re-ChIP experiments were performed to determine whether ZKSCAN5 was associated with SETD7 on the −658- to −608-bp region of the VEGFC promoter (**Figure 5A**). Importantly, ZKSCAN5 knockdown reduced the recruitment of SETD7, H3K4me2, and H3K4me3, to the −658- to −608-bp region of the VEGFC promoter (**Figure 5B**). Knockout of SETD7 reduced the recruitment of H3K4me2 and H3K4me3 to the −658- to −608-bp region of the VEGFC promoter (**Figure 5B**). The same trend was observed by using (R)-PFI-2 (1 μM, 2 h), an inhibitor of SETD7 (**Figure 5C**).

Based on the fact that ZKSCAN5 could mediate the enrichment of SETD7, we investigated whether ZKSCAN5 could substantially interact with SETD7. Endogenous ZKSCAN5 pointedly coimmunoprecipitated with endogenous SETD7 using ZR75-1 cells (**Figure 5D**). Since the His-labelled ZKSCAN5 protein interacts with the purified GST-SETD7, but not GST alone, the functional interaction between ZKSCAN5 and SETD7 is explicit (**Figure 5E**). ZKSCAN5 (215–366) contains the SCAN domain related to Set7 but does not contain other ZKSCAN5 deletion mutants (**Figure 5F**). SET7 (215–366) contains the SET fragment (SET), which interacted with ZKSCAN5, whereas SET7 (108–214) containing the middle-region fragment (MF) and SET7 N-terminal region (1–107) containing the NF domain did not (**Figure 5G**). These results show that ZKSCAN5 and SETD7 may construct complexes in the −658- to −608-bp region of the VEGFC promoter.

### ZKSCAN5 Positively Correlates With VEGFC Expression and Plays a Prognostic Role in Breast Cancer

**Figure 6:**
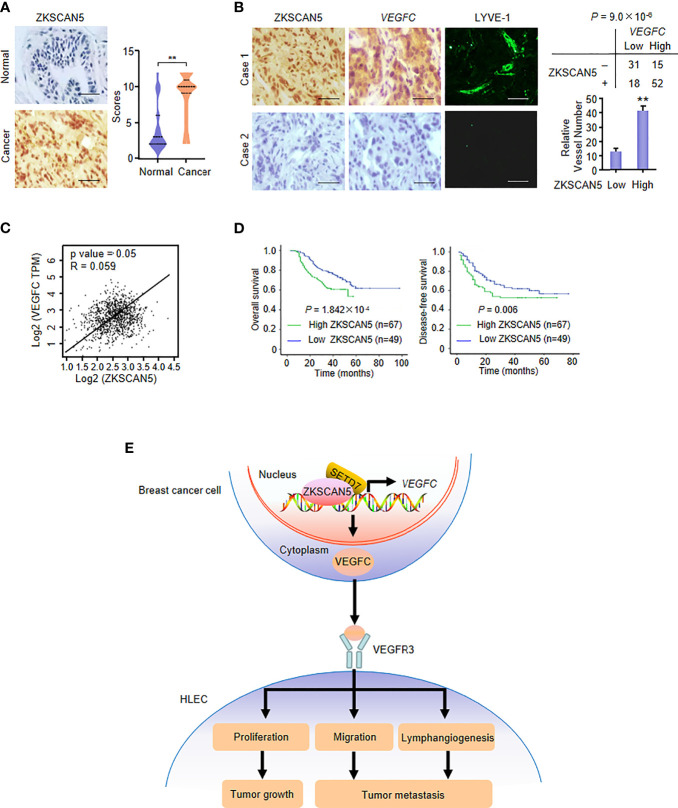
ZKSCAN5 is a prognostic marker of breast cancer and is positively correlated with *VEGFC* expression. **(A)** Representative immunohistochemical staining of ZKSCAN5 in human cancerous breast tissues and adjacent normal breast tissues. Scale bar: 25 μm. ZKSCAN5 expression scores were plotted and compared (Mann–Whitney *U* test). **(B)** Representative immunohistochemical staining of ZKSCAN5 in human breast cancer samples. Scale bar: 25 μm. To quantify lymphatic microvessel density, images were obtained from eight regions of each tissue to calculate the number of vessels more accurately. The correlation of ZKSCAN5 with *VEGFC* expression or lymph microvessel number (positive LYVE-1 staining) is shown. The p value was generated using Pearson’s χ^2^ test (ZKSCAN5 and *VEGFC*) and the Wilcoxon rank-sum test (LYVE-1). **(C)** Database analysis showed that ZKSCAN5 expression was positively correlated with VEGFC expression in breast cancer patients. **(D)** The total survival time (above) and disease-free survival time (below) of patients with breast cancer were estimated by the Kaplan–Meier method. The review samples are represented by the markers on the chart lines. **(E)** Proposed model for ZKSCAN5 modulation of VEGFC expression as well as its tumour-promoting function. ZKSCAN5 recruited SETD7 into the VEGFC promoter, which promoted the increase of VEGFC transcription and secretion in breast cancer cells. VEGFC is secreted by cancer cells, binding to VEGFR3, promoting proliferation, migration and lymphangiogenesis of HELC. Ultimately, they can lead to tumour growth and metastasis.

We first performed immunohistochemistry (IHC) on 116 human breast cancer samples to demonstrate the clinical significance of ZKSCAN5. Before this test, the specificity of the antibodies for ZKSCAN5 in IHC was determined by immunoblotting lysates from MDA-MB-231 and ZR75-1 breast cancer cells transfected with ZKSCAN5 siRNAs (**Supplementary Figure S2**). Interestingly, ZKSCAN5 expression increased in cancer tissues compared to that in the adjacent paracancerous tissues (p = 2.33 × 10^−6^; **Figure 6A**). The associations between ZKSCAN5 expression and lymph vessel number stained by the specific marker LYVE-1 were investigated. ZKSCAN5 was positively related to VEGFC expression in breast cancer tissues (p = 9.0 × 10^−6^). Tumours with high ZKSCAN5 expression had more lymph vessels compared with low ZKSCAN5 expression (**Figures 6B, C** **)**. Moreover, we observed that higher ZKSCAN5 expression indicated reduced disease-free (p = 1.842 × 10^−4^) and overall survival (p = 0.006; **Figure 6D**). In conclusion, these findings imply the importance of ZKSCAN5 in lymphangiogenesis and the prognosis of breast cancer.

## Discussion

Lymphatic vasculature is considered a crucial factor in the modulation of normal homeostasis and many diseases (24). VEGFC is one of the most important regulators of tumour lymphangiogenesis. Emerging evidence shows that various aspects of tumour development can be promoted through the autocrine regulation of VEGFC. It is reported that VEGFC can also regulate the immune system, making it easier for tumour cells to escape immune surveillance. The proliferation and migration of lymphatic endothelial cells are prerequisites for lymphangiogenesis (25). The expression of VEGFD in breast tumours was significantly higher than that in the non-adjacent control (26, 27). The expression of VEGFC was significantly higher than that of VEGFD in patients with breast cancer, as revealed by investigating TCGA database (**Supplementary Figure S3**, p < 0.0001). A study demonstrated that primary breast tumours induce sentinel lymph node lymphangiogenesis and that tumour-derived VEGFC plays an important role in their lymphangiogenesis in breast cancer, but not VEGFD (28). VEGFD seemed to exert proliferative activity in invasive breast carcinomas. VEGFC was found to be an independent indicator of a patient’s poor prognosis (29). Thus, elucidating the molecular mechanisms underlying VEGFC expression modulation in cancer cells is of great significance.

The significant upregulation and downregulation of VEGFC expression in tumours were mainly caused by transcriptional regulation (30). Transcription factors, such as Six1 (13) and FOXK1 (14), enhanced VEGFC transcription among cancer cells. However, other transcriptional factors that regulate VEGFC expression remain largely unknown. Here, we identified ZKSCAN5 as a novel transcriptional factor for VEGFC expression regulation. We chose ZR75-1 and MDA-MB-231 breast cancer cell lines to exclude the influence of the ER status. ZKSCAN5 can not only activate the activity of the VEGFC-Luc reporter but also increase the expression of VEGFC mRNA. ZKSCAN5 was localised predominantly in the nucleus, which provided the cellular basis of ZKSCAN5 to regulate VEGFC expression transcriptionally. ZKSCAN5 binds to the promoter section (−2,911 to −2,859 bp) of VEGFC in breast cancer cells. Cancer cell-secreted VEGFC regulated by ZKSCAN5 controls HLEC proliferation, migration, and tube formation (**Figure 6E**). As a new clinical prognostic marker for breast cancer, ZKSCAN5 has a positive correlation with VEGFC expression. Thus, targeting ZKSCAN5 will be an effective way to control lymphangiogenesis in breast cancer.

Transcriptional regulation consists of changes in transcription factor binding and a complex programme of epigenetic changes regulated by histone-modifying enzymes and DNA methyltransferase (31, 32). However, the characteristics of transcription factor binding-related histone modification enzymes remain unclear. Unlike many other methyltransferases, SETD7 only monomethylates H3K4, resulting in transcription activation in HeLa cells (33). SETD7 has initially been defined as H3K4me1. It remains controversial whether SETD7-mediated H3K4me1 is critical for the transcriptional regulation of its target genes (34). Although SETD7 is one H3K4-specific methyltransferase, SETD7-mediated p53 methylation is not a major regulatory event and does not affect p53 activity markedly *in vivo* (35). Although Guo et al. established the physical interaction between ISL1 and SETD7, as a histone H3K4-specific methyltransferase (36), SETD7 activates its expression in gastric cancer cells by binding to the ZEB1 promoter (37). Despite SETD7 being generally considered a monomethyltransferase, it has also been shown to catalyse the dimethylation of specific substrates, depending on the sequence contexts of the methylation sites (38). For example, researchers discovered that SETD7-mediated H3K4me3 enrichment on the lncRNA DRAIC promoter regulated the growth and metastasis of gliomas (39). We confirmed a functional role for ZKSCAN5 in recruiting SETD7 to the specific target VEGFC. ZKSCAN5 directly interacts with SETD7 and forms a complex with SETD7 on the VEGFC promoter. ZKSCAN5 knockdown reduces the recruitment of SETD7, H3K4me2, and H3K4me3. SETD7 knockdown or inhibition decreases H3K4me2 and H3K4me3 expression on the VEGFC promoter. Notably, SETD7 is known to be a transcriptional coactivator for ZKSCAN5 in regulating VEGFC transcription. Our study showed that SETD7 could play an important role in the transcriptional regulation of ZKSCAN5 as a cofactor of H3K4me2 or H3K4me3. The different results from previous studies may be caused by the following factors: first, previous studies have focused on different cell lines; second, many factors can affect the target genes excluding DNA methylation; third, sequence contexts of the methylation sites could lead to different outcomes. Taken together, our study indicates the critical role of ZKSCAN5 in epigenetic regulation and suggests that methylation of H3K4me2 and H3K4me3 by SETD7 is required for ZKSCAN5-induced VEGFC transcription.

ZKSCAN5 is proposed to play an important role during spermatogenesis (15). In humans, alternatively spliced ZKSCAN5 transcripts with different 5′-untranslated regions have been confirmed (40). However, the biological function of ZKSCAN5 is currently largely unknown. To the best of our knowledge, here, for the first time, we uncovered the function of ZKSCAN5 in modulating VEGFC expression, lymphangiogenesis, and breast cancer cell growth. In addition, we found that ZKSCAN5 overexpression was positively correlated with a poor prognosis in patients with breast cancer. ZKSCAN5 is the first identified sequence-specific DNA-binding transcription factor that can bind to the VEGFC promoter (**Figure 4D**). Our results pertaining to ZKSCAN5 supplement previous findings of the biological functions of ZKSCAN5. However, there have been few relevant studies on ZKSCAN5 since its discovery in 1999 (41). ZKSCAN3, a transcription factor in the same family as ZKSCAN5, plays a role in many types of tumours (42–44). ZKSCAN3 is a zinc finger transcription factor with KRAB and SCAN domains. It upregulates the expression of genes related to the cell cycle, resulting in cell proliferation, migration, angiogenesis, and proteolysis. Therefore, ZKSCAN3 promotes the tumour progression, invasion, and migration and cell growth. Silencing its expression can significantly suppress the malignancy, tumorigenicity of xenotransplantats, and growth and metastasis of tumour cells. Knocking out this key molecule in tumour cells can also lead to the enhancing of the antitumor effects of drugs. The wide expression of ZKSCAN3 in tumour cells makes it an important potential target for tumour therapy. ZKSCAN5 and ZKSCAN3 belong to the zinc finger transcription factor family. We propose that their functions would also be similar and, along with ZKSCAN3, can be targeted for the development of tumour therapy.

It has been reported that SETD7 methylates ERα, which plays an important role in breast cancer development and progression (45). Recently, SETD7 has been found to potentially methylate β-catenin, which plays a key role in cytodifferentiation, cell proliferation, and tumorigenesis (46). Inactivation or elimination of SETD7 causes G1/S cell-cycle arrest in osteosarcoma and pulmonary carcinoma cells after DNA damage (47, 48). It remains to be explored whether SETD7 has tissue-specific effects on regulating the growth of cancer cells. The present study provides some evidence that SETD7 is an oncogene in breast cancer. Inhibiting SETD7 expression may be a good strategy for breast cancer treatment. It is very interesting to study these inhibitors that may restrain tumour cell growth and lymphangiogenesis.

## Data Avalability Statement

The datasets presented in this study can be found in online repositories. The names of the repository/repositories and accession number(s) can be found in the article/**Supplementary Material**.

## Ethics Statement

The studies involving human participants were reviewed and approved by the Ethics Committee of Harbin Medical University Cancer Hospital. The patients/participants provided their written informed consent to participate in this study. The animal study was reviewed and approved by the Ethics Committee of Harbin Medical University Cancer Hospital. Written informed consent was obtained from the individual(s) for the publication of any potentially identifiable images or data included in this article.

## Author Contributions

QZ, HZ, YL and TW conceived the idea of the study; JL, ZY and JM analysed the data; ZC, HL, JG interpreted the results; JL, ZY and JM wrote the paper; all authors discussed the results and revised the manuscript.

## Funding

This study was supported by the National Natural Science Foundation of China [grant number 81730074].

## Conflict of Interest

The authors declare that the research was conducted in the absence of any commercial or financial relationships that could be construed as a potential conflict of interest.

## Publisher’s Note

All claims expressed in this article are solely those of the authors and do not necessarily represent those of their affiliated organizations, or those of the publisher, the editors and the reviewers. Any product that may be evaluated in this article, or claim that may be made by its manufacturer, is not guaranteed or endorsed by the publisher.
