# Supplementary material for: ZKSCAN5 Activates VEGFC Expression by Recruiting SETD7 to Promote the Lymphangiogenesis, Tumour Growth, and Metastasis of Breast Cancer
Source: Front Oncol. 2022 May 5;12:875033. doi: 10.3389/fonc.2022.875033 (PMC9117617; doi:10.3389/fonc.2022.875033)
Supplement: Supplementary file 1 [file DataSheet_1.pdf]

Figure S1

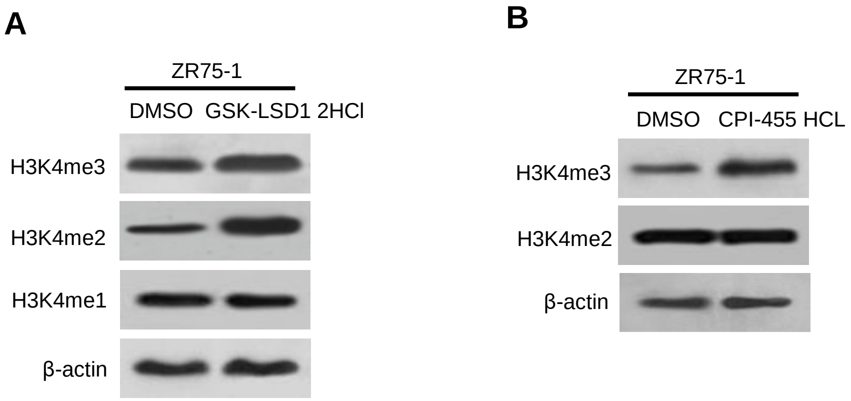

Supplementary Figure 1. Validation of the specificity of H3K4methyl antibodies used in WB. (A) Immunoblot analysis of lysates from ZR75-1 cells treated with DMSO or GSK-LSD1 2HCL. (B) Immunoblot analysis of lysates from ZR75-1 cells treated with DMSO or CPI-455 HCL. Data shown are mean ± SD of triplicate measurements and have been repeated 3 times with similar results.

Figure S2

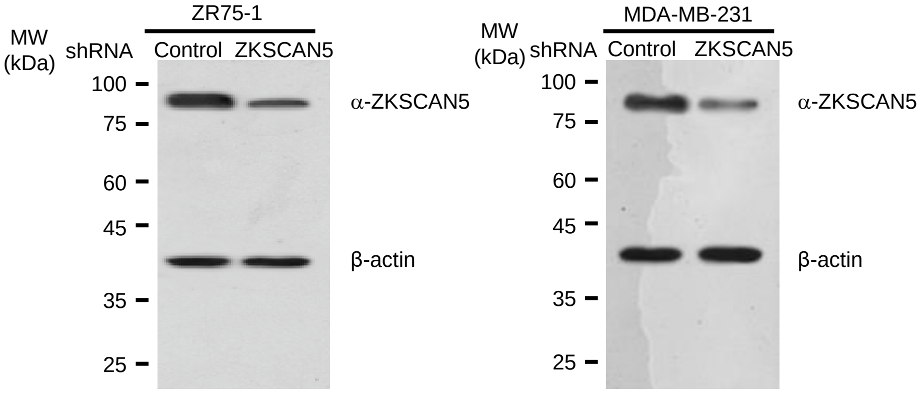

Supplementary Figure 2. Identification of the specificity of ZKSCAN5 antibody used in WB. Immunoblot analysis of lysates from ZR75-1 or MDA-MB-231 cells infected with control shRNA or ZKSCAN5 shRNA. Data shown are mean ± SD of triplicate measurements and have been repeated 3 times with similar results.

Figure S3

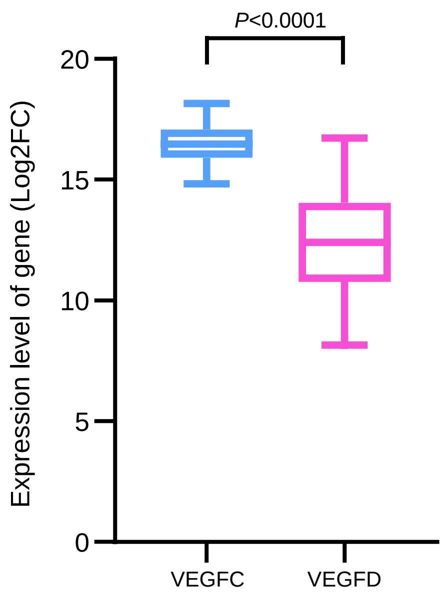

Supplementary Figure 3. VEGFC and VEGFD expression in BC patients revealed by the TCGA database (*P*<0.0001).
