# Supplementary material for: ZKSCAN5 Activates VEGFC Expression by Recruiting SETD7 to Promote the Lymphangiogenesis, Tumour Growth, and Metastasis of Breast Cancer
Source: Front Oncol. 2022 May 5;12:875033. doi: 10.3389/fonc.2022.875033 (PMC9117617; doi:10.3389/fonc.2022.875033)
Supplement: Supplementary file 2 [file Table_1.docx]

**Table.1 Primers used** **sequences for real-time PCR**

| ZKSCAN5-F | GCAGAGGCTTCACTCTGAAGTCACATC |
| --- | --- |
| ZKSCAN5-R | GGATCTGTCCTCTCATGGCTTCTCAG |
| VEGFC-F | CTCTCTCAAGGCCCCAAACC |
| VEGFC-R | TCTTGTTCGCTGCCTGACACT |
| β-actin-F | ATCACCATTGGCAATGAGCG |
| β-actin-R | TTGAAGGTAGTTTCGTGGAT |

**Table 2. The cDNA target sequences of shRNAs or siRNAs**

| SETD7 siRNA1/shRNA1 | GGTTTATGTTGCCGACTC |
| --- | --- |
| SETD7 siRNA2/shRNA2 | GGTAGCAGTTGGACCTAAT |
| ZKSCAN5 siRNA/shRNA | CCCTCTGAGTTGGAATGAT |

**Table 3. Primers used for ChIP**

| VEGFC Promoter-F | TCCTCTGTAACCTGCTCACCCTGCC |
| --- | --- |
| VEGFC Promoter-R | CCTCCCCTTCCCCGAAGTGAG |
| VEGFC upstream-F | GTTAAAAATATAAACAAATTAAG |
| VEGFC upstream-R | CGGGCCATGT GTGGTGGCTC |
| VEGFC Promoter(-658~-608)-F | CTGACATAGTGATGACCTTTTC |
| VEGFC Promoter(-658~-608)-R | AATGTCCTGGGGGTTGTAAAAG |
| VEGFC Promoter(-608~-558)-F | TCCAAACTTTGAGCAGGGCTC |
| VEGFC Promoter(-608~-558)-R | TGTCCTCCCT CCCGCACGCC |
